# Acknowledgement of manuscript reviewers 2015

**DOI:** 10.1186/s12966-016-0347-0

**Published:** 2016-02-17

**Authors:** Russell Jago

**Affiliations:** University of Bristol, Bristol, UK

## Abstract

The editors of *International Journal of Behavioral Nutrition and Physical Activity* would like to thank all our reviewers who have contributed to the journal in Volume 12 (2015).

**Adam Walsh**

Australia

**Adrian Cameron**

Australia

**Adrian Davis**

United Kingdom

**Aileen Grant**

United Kingdom

**Alison Carver**

Australia

**Alison Miller**

United States of America

**Alison Venn**

Australia

**Allison Watts**

United States of America

**Amanda Birnbaum**

United States of America

**Amanda Lee**

Australia

**Amanda Patterson**

Australia

**Amanda Staiano**

United States of America

**Amber Vaughn**

United States of America

**Amy Mcpherson**

Canada

**Ana Baylin**

United States of America

**Anders Grøntved**

Denmark

**Andreas Holtermann**

Denmark

**Andrew J Atkin**

United Kingdom

**Andrew Springer**

United States of America

**Andy Jones**

United Kingdom

**Angela Donin**

United Kingdom

**Angeliki Papadaki**

United Kingdom

**Anita Hurtig-Wennlof**

Sweden

**Anne Loyen**

Netherlands

**Annemarie Koster**

Netherlands

**Annie Anderson**

United Kingdom

**Anouk Middelweerd**

Netherlands

**Anthony Okely**

Australia

**April Carcone**

United States of America

**April Idalski Carcone**

United States of America

**Asa Tornberg**

Sweden

**Ashley Gearhardt**

United States of America

**Astrid Etman**

Netherlands

**Austin Baldwin**

United States of America

**Barbara Ainsworth**

United States of America

**Barbara Fiese**

United States of America

**Benedicte Deforche**

Belgium

**Benjamin Gardner**

United Kingdom

**Bente Wold**

Norway

**Birgit Wallmann-Sperlich**

Germany

**Bongers Peggy**

Netherlands

**Brandon Irwin**

United States of America

**Bridget Kelly**

Australia

**Bronwyn Clark**

Australia

**Callie Brown**

United States of America

**Carlos Monterio**

Brazil

**Carol Byrd-Bredbenner**

United States of America

**Carol Maher**

Australia

**Catherine Chan**

Canada

**Catherine Draper**

South Africa

**Catherine Pacquet**

Australia

**Catherine Russell**

Australia

**Catherine Woods**

Ireland

**Catrine Tudor-Locke**

United States of America

**Charlie Foster**

United Kingdom

**Chris Rissel**

Australia

**Christine Blake**

United States of America

**Christine Fekete**

Switzerland

**Cindy Gray**

United Kingdom

**Ciska Hoving**

Netherlands

**Claire Bernaards**

Netherlands

**Claudia Vadeboncoeur**

United Kingdom

**Colin Greaves**

United Kingdom

**Colin Rehm**

United States of America

**Corneel Vandelanotte**

Australia

**Dafna Merom**

Australia

**Dan Bornstein**

United States of America

**Dana Lee Olstad**

Australia

**Dane Van Domelen**

United States of America

**Daniel Aggio**

United Kingdom

**David Berrigan**

United States of America

**David Cavallo**

United States of America

**David Crawford**

Australia

**David Dzewaltowski**

United States of America

**David Lubans**

Australia

**David Mcminn**

United Kingdom

**Debbe Thompson**

United States of America

**Debbie Ann Loh**

Malaysia

**Deborah Kerr**

Australia

**Deirdre Harrington**

United Kingdom

**Delfien Van Dyck**

Belgium

**Diane Ehlers**

United States of America

**Diane Jackson**

United Kingdom

**Dianne Ward**

United States of America

**Dori Rosenberg**

United States of America

**Dorota Zarnowiecki**

Australia

**Dorus Gevers**

Netherlands

**Duncan Macfarlane**

Hong Kong

**Edward Coffield**

United States of America

**Elin Johansson**

Sweden

**Elissa Jelalian**

United States of America

**Elizabeth Eakin**

Australia

**Elling Bere**

Norway

**Emily Kothe**

Australia

**Emma Boyland**

United Kingdom

**Emma Coombes**

United Kingdom

**Emma Foster**

United Kingdom

**Emma Lea**

Australia

**Emma Thomas**

United Kingdom

**Enrique Garcia Bengoechea**

Canada

**Eric Stice**

United States of America

**Eric Wickel**

United States of America

**Erica Hinckson**

New Zealand

**Erica James**

Australia

**Erica L. Kenney**

United States of America

**Erik Hemmingsson**

Sweden

**Erin Hennessy**

United States of America

**Erin Mead**

United States of America

**Erin Roche**

United States of America

**Ester Cerin**

United States of America

**Ester Sleddens**

Netherlands

**Esther Van Sluijs**

United Kingdom

**Fabiola Aparicio-Ting**

Canada

**Fariba Kolahdooz**

Canada

**Farid Bardid**

Belgium

**Faryle Nothwehr**

United States of America

**Femke Van Nassau**

Netherlands

**Ferdinand Salonna**

Czech Republic

**Fiona Bull**

Australia

**Fiona Bull**

United Kingdom

**Flo Harrison**

United Kingdom

**Frank Van Lenthe**

Netherlands

**Gail Regan**

United States of America

**Gavin Abbott**

Australia

**Gavin Turrell**

Australia

**Genevieve Healy**

Australia

**Gerry Jager**

Netherlands

**Gill Cowburn**

United Kingdom

**Gisela Nyberg**

Sweden

**Graham Baker**

United Kingdom

**Greet Cardon**

Belgium

**Gregory Heath**

United States of America

**Gretchen Cutler**

United States of America

**Hayley Christian**

Australia

**Heather Kitzman-Ulrich**

United States of America

**Helen Elizabeth Brown**

United Kingdom

**Helene Charreire**

France

**Ian Cook**

South Africa

**Ian Janssen**

Canada

**Ian Kellar**

United Kingdom

**Idia Thurston**

United States of America

**Ilse De Bourdeaudhuij**

Belgium

**Ingrid Steenhuis**

Netherlands

**Jacob Barkley**

United States of America

**Jacqueline Kerr**

United States of America

**Jane Lanigan**

United States of America

**Jane Wardle**

United Kingdom

**Janet Hauck**

United States of America

**Janet Withall**

United Kingdom

**Jantine Schuit**

Netherlands

**Jason Fanning**

United States of America

**Jason Mendoza**

United States of America

**Jasper Schipperijn**

Denmark

**Jayne Fulkerson**

United States of America

**Jean Adams**

United Kingdom

**Jeanette Garcia**

United States of America

**Jean-Michel Oppert**

France

**Jeanne De Vries**

Netherlands

**Jeffrey Labban**

United States of America

**Jelle Van Cauwenberg**

Belgium

**Jenna Hollis**

United Kingdom

**Jennette Moreno**

United States of America

**Jennifer Linde**

United States of America

**Jennifer O'Neill**

United States of America

**Jennifer Pomeranz**

United States of America

**Jennifer Robertson-Wilson**

Canada

**Jenny Veitch**

Australia

**Jens Bucksch**

Germany

**Jeroen Lakerveld**

Netherlands

**Jessica Herbert**

Australia

**Jessica Jones-Smith**

United States of America

**Jesus Soares**

United States of America

**Jill Hnatiuk**

Australia

**Jo Kesten**

United Kingdom

**Jo Salmon**

Australia

**Johan Eriksson**

Finland

**John Spence**

Canada

**Jonathan Mitchell**

United States of America

**Joreintje Mackenbach**

Netherlands

**Jorge Mota**

Portugal

**Josef Mitas**

Czech Republic

**Josephine Chau**

Australia

**Josephine Gwynn**

Australia

**Josie Booth**

United Kingdom

**Jostein Steene-Johannessen**

Norway

**Jouni Lahti**

Finland

**Juan Fernández-Alvira**

Spain

**Julia Alber**

United States of America

**Julie Obbagy**

United States of America

**Julie Obbagy**

United States of America

**Julie Obbagy**

United States of America

**Julie Obbagy**

United States of America

**Justin Brown**

United States of America

**Justin Brown**

United States of America

**Justin Brown**

United States of America

**Justin Richards**

Australia

**Jyh Eiin Wong**

Malaysia

**Kaigang Li**

United States of America

**Karen Cullen**

United States of America

**Karen Cullen**

United States of America

**Karen Cullen**

United States of America

**Karen Glanz**

United States of America

**Karen Glanz**

United States of America

**Karen Glanz**

United States of America

**Karen Milton**

United Kingdom

**Karen Milton**

United Kingdom

**Karin Bammann**

Germany

**Kate Hunt**

United Kingdom

**Katherine Bauer**

United States of America

**katherine bauer**

United States of America

**Katherine Hanna**

Australia

**Kathleen Lacy**

Australia

**Kathleen Lacy**

Australia

**Katja Siefken**

Germany

**Katrien Wijndaele**

United Kingdom

**Katrien Wijndaele**

United Kingdom

**Kelly Evenson**

United States of America

**Kelly Evenson**

United States of America

**Kelly Evenson**

United States of America

**Kerith Duncanson**

Australia

**Kerith Duncanson**

Australia

**Kim Gans**

United States of America

**Kim Gans**

United States of America

**Kim Gans**

United States of America

**Kimmo Sorjonen**

Sweden

**Kirsten Corder**

United Kingdom

**Klaus Gebel**

Australia

**Klaus Gebel**

Australia

**Knut-Inge Klepp**

Norway

**Knut-Inge Klepp**

Norway

**Kylie Ball**

Australia

**Kylie Hesketh**

Australia

**Kylie Hesketh**

Australia

**Kylie Hesketh**

Australia

**Kylie Smith**

Australia

**Kylie Smith**

Australia

**Kyung Rhee**

United States of America

**Laura Johnson**

United Kingdom

**Laura Johnson**

United Kingdom

**Lauren Rossen**

United States of America

**Lawrence Foweather**

United Kingdom

**Leah Robinson**

United States of America

**Leigh Gibson**

United Kingdom

**Lekki Wood**

United States of America

**Leonie Uijtdewilligen**

Singapore

**Leonie Uijtdewilligen**

Singapore

**Lilian Lechner**

Netherlands

**Lin Lin**

China

**Lisa Phillips**

United Kingdom

**Lisa Phillips**

United Kingdom

**Liselotte Schäfer Elinder**

Sweden

**Liselotte Schäfer Elinder**

Sweden

**Liselotte Schäfer Elinder**

Sweden

**Liselotte Schäfer Elinder**

Sweden

**Lori Andersen**

United States of America

**Louise C Masse**

Canada

**Louise Foley**

United Kingdom

**Lucy Cooke**

United Kingdom

**LUIS GRACIA-MARCO**

United Kingdom

**Luis Sardinha**

Portugal

**Lukar Thornton**

Australia

**Lukar Thornton**

Australia

**Lukar Thornton**

Australia

**Luke Wolfenden**

Australia

**Luke Wolfenden**

Australia

**Lydia Kwak**

Sweden

**Lydia Kwak**

Sweden

**Lydia Kwak**

Sweden

**Lynn Edmunds**

United States of America

**Lynsay Matthews**

United Kingdom

**Lynsey Smith**

United States of America

**Lynsey Smith**

United States of America

**Mai Chinapaw**

Netherlands

**Maite Verloigne**

Belgium

**Mandy Ho**

Hong Kong

**Margaret Allman-Farinelli**

Australia

**Margaret Allman-Farinelli**

Australia

**Maria Hagstromer**

Sweden

**Marieke Antonia Hartman**

United States of America

**Marjolein Visser**

Netherlands

**Marjorie Freedman**

United States of America

**Mark Hamer**

United Kingdom

**Marla Reicks**

United States of America

**Matthew Buman**

United States of America

**Meg bruening**

United States of America

**Megan Jarman**

Canada

**Megan Rollo**

Australia

**Megan Teychenne**

Australia

**Megan Teychenne**

Australia

**Meizi He**

United States of America

**Melanie Hingle**

United States of America

**Melanie Hingle**

United States of America

**Melanie Hingle**

United States of America

**Melinda Hutchesson**

Australia

**Melissa Horning**

United States of America

**Melvyn Hillsdon**

United Kingdom

**Melvyn Hillsdon**

United Kingdom

**Melvyn Hillsdon**

United Kingdom

**Melvyn Hillsdon**

United Kingdom

**Melvyn Hillsdon**

United Kingdom

**MinKyoung Song**

United States of America

**Minna Aittasalo**

Finland

**Minna Aittasalo**

Finland

**Mitch J Duncan**

Australia

**Moira Dean**

United Kingdom

**Nana Anokye**

United Kingdom

**Nanna Lien**

Norway

**Nanna Lien**

Norway

**Nanna Lien**

Norway

**Nanna Lien**

Norway

**Nanna Lien**

Norway

**Natalie Pearson**

United Kingdom

**Natalie Pearson**

United Kingdom

**Natalie Pearson**

United Kingdom

**Natalie Pearson**

United Kingdom

**Nicholas Gilson**

Australia

**Nicola Diane Ridgers**

Australia

**Nicola Ridgers**

Australia

**Nicola Ridgers**

Australia

**Nicole Larson**

United States of America

**Niko Kaciroti**

United States of America

**Niko Kaciroti**

United States of America

**Niko Kaciroti**

United States of America

**Niko Kaciroti**

United States of America

**Niko Kaciroti**

United States of America

**Niko Kaciroti**

United States of America

**Noe Crespo**

United States of America

**Örjan Ekblom**

Sweden

**Örjan Ekblom**

Sweden

**Patricia Hageman**

United States of America

**Patricia Hageman**

United States of America

**Patricia Hageman**

United States of America

**Patrick Bergman**

Sweden

**Patti-Jean Naylor**

Canada

**Patti-Jean Naylor**

Canada

**Paul Fuglestad**

United States of America

**Paul Gardiner**

Australia

**Paul Kelly**

United Kingdom

**Paul Loprinzi**

United States of America

**Pauliina Husu**

Finland

**Paulina Nowicka**

Sweden

**Pauline Jansen**

Netherlands

**Pedro Teixeira**

Portugal

**Pedro Teixeira**

Portugal

**Pedro Teixeira**

Portugal

**Peter Hannan**

United States of America

**Peter Hannan**

United States of America

**Peter Katzmarzyk**

United States of America

**Philip Baker**

Australia

**Philip Troped**

United States of America

**Prachi Bhatnagar**

United Kingdom

**Rachel Pechey**

United Kingdom

**Ralph Maddison**

New Zealand

**Ralph Maddison**

New Zealand

**Ralph Maddison**

New Zealand

**Ralph Maddison**

New Zealand

**Rebecca Golley**

Australia

**Rebecca Leech**

Australia

**Rebecca Leech**

Australia

**Rebecca Leech**

Australia

**Rebecca Wyse**

Australia

**Richard G. Prins**

Netherlands

**Richard G. Prins**

Netherlands

**Richard G. Prins**

Netherlands

**Richard Larouche**

Canada

**Richard Mitchell**

United Kingdom

**Richard Rosenkranz**

United States of America

**Richard Rosenkranz**

United States of America

**Richard Rosenkranz**

United States of America

**Richard Rosenkranz**

United States of America

**Richard Troiano**

United States of America

**Robert W. Jeffery**

United States of America

**Robert W. Jeffery**

United States of America

**Robert W. Jeffery**

United States of America

**Robert W. Jeffery**

United States of America

**Robin Callister**

Australia

**Robin Callister**

Australia

**Robin Mellecker**

Hong Kong

**Ronald Plotnikoff**

Australia

**Ronald Plotnikoff**

Australia

**Ronald Plotnikoff**

Australia

**Ruth Hunter**

United Kingdom

**Ruth Kipping**

United Kingdom

**Ruth Mabry**

Oman

**Ruth Mabry**

Oman

**Ruth Mabry**

Oman

**Ruth Mabry**

Oman

**Ryan Rhodes**

Canada

**Ryan Rhodes**

Canada

**Sally Akarolo-Anthony**

United States of America

**Sally Mackay**

New Zealand

**Sandar Tin Tin**

New Zealand

**Sandar Tin Tin**

New Zealand

**Sandra Mandic**

New Zealand

**Sandy Slater**

United States of America

**Sandy Slater**

United States of America

**Sanford DeVoe**

Canada

**Sanne Gerards**

Netherlands

**Sanne Gerards**

Netherlands

**Sarah Kozey Keadle**

United States of America

**Sarah Mcnaughton**

Australia

**Sarah McNaughton**

Australia

**Scott Duncan**

New Zealand

**Scott Duncan**

New Zealand

**Scott Duncan**

New Zealand

**Scott Duncan**

New Zealand

**Scott Duncan**

New Zealand

**Scott Duncan**

New Zealand

**Scott Duncan**

New Zealand

**Scott Duncan**

New Zealand

**Sebastien Chastin**

United Kingdom

**Sebastien Chastin**

United Kingdom

**Shayla Holub**

United States of America

**Shayla Holub**

United States of America

**Sheryl Hughes**

United States of America

**Shigeru Inoue**

Japan

**Shigeru Inoue**

Japan

**Shigeru Inoue**

Japan

**Shufa Du**

United States of America

**Shufa Du**

United States of America

**Shufa Du**

United States of America

**Shufa Du**

United States of America

**Shufa Du**

United States of America

**Shufa Du**

United States of America

**Shufa Du**

United States of America

**Shufa Du**

United States of America

**Shufa Du**

United States of America

**Shufa Du**

United States of America

**Shufa Du**

United States of America

**Sibylle Kranz**

United Kingdom

**Sibylle Kranz**

United Kingdom

**Simon Sebire**

United Kingdom

**Simon Sebire**

United Kingdom

**Simon Sebire**

United Kingdom

**Simone Dohle**

Germany

**Simone Dohle**

Germany

**Simone Dohle**

Germany

**Simone Dohle**

Germany

**Simone Pettigrew**

Australia

**Sonja Kahlmeier**

Switzerland

**Stef Kremers**

Netherlands

**Stef Kremers**

Netherlands

**Stefanie Vandevijvere**

New Zealand

**Stefanie Vandevijvere**

New Zealand

**Stephanie Chambers**

United Kingdom

**Stephanie Schoeppe**

Australia

**Stephanie Schoeppe**

Australia

**Stephenie Lemon**

United States of America

**Steve Herrmann**

United States of America

**Stuart Biddle**

Australia

**Stuart Biddle**

Australia

**Stuart Biddle**

Australia

**Stuart Biddle**

Australia

**Stuart Biddle**

Australia

**Stuart Biddle**

Australia

**Stuart Biddle**

Australia

**Stuart Biddle**

Australia

**Stuart Biddle**

Australia

**Stuart Biddle**

Australia

**Stuart Fairclough**

United Kingdom

**Susan Carnell**

United States of America

**Susan Carnell**

United States of America

**Susan Carnell**

United States of America

**Susan Handy**

United States of America

**Susan Paxton**

Australia

**Susan Slade**

Australia

**Susan Slade**

Australia

**Suzanna Forwood**

United Kingdom

**Suzanne Domel Baxter**

United States of America

**Suzanne Domel Baxter**

United States of America

**Sylvia Titze**

Austria

**Sylvia Titze**

Austria

**Sylvia Titze**

Austria

**Sylvia Titze**

Austria

**Tamara Bucher**

Australia

**Tamara Bucher**

Australia

**Tamara Bucher**

Australia

**Tamara Bucher**

Australia

**Tamara Bucher**

Australia

**Tanya Berry**

Canada

**Teatske Altenburg**

Netherlands

**Teresia O'Connor**

United States of America

**Teresia O'Connor**

United States of America

**Thomas Power**

United States of America

**Thomas Power**

United States of America

**Thomas Power**

United States of America

**Thomas Power**

United States of America

**Thomas Power**

United States of America

**Tiago Barreira**

United States of America

**Tom Baranowski**

United States of America

**Tom Baranowski**

United States of America

**Tom Baranowski**

United States of America

**Tom Baranowski**

United States of America

**Tom Baranowski**

United States of America

**Tracy Kolbe-Alexander**

Australia

**Tracy Richmond**

United States of America

**Trina Hinkley**

Australia

**Trinidad Quizan**

Mexico

**Tuija Tammelin**

Finland

**Tzu-An Chen**

United States of America

**Vera Storm**

Germany

**Victor Viana**

Portugal

**Virginia Quick**

United States of America

**Wendy Van Lippevelde**

Belgium

**Wendy Van Lippevelde**

Belgium

**Wendy Van Lippevelde**

Belgium

**Wim Grooten**

Sweden

**xiaoli chen**

United States of America

**Yang Liu**

Finland

**Yang Liu**

Finland

**Youngwon Kim**

United Kingdom

**Zan Gao**

United States of America

**Zan Gao**

United States of America

**Zewditu Demissie**

United States of America

